# Comprehensive profiling of Alzheimer's disease in Kazakhstan: clinical, genetic, and microbiome insights

**DOI:** 10.1002/alz70855_097791

**Published:** 2025-12-23

**Authors:** Aiym Kaiyrlykyzy, Gulnaz Zholdasbekova, Dinara Alzhanova, Samat Kozhakhmetov, Almagul Kushugulova, Sholpan Askarova

**Affiliations:** ^1^ Al‐Farabi Kazakh National University, Almaty, Kazakhstan; ^2^ Center for Life Sciences, National Laboratory Astana, Nazarbayev University, Astana, Kazakhstan; ^3^ Medical University Karaganda, Karaganda, Kazakhstan; ^4^ Astana Medical University, Astana, Kazakhstan; ^5^ Kazakhstan society of human microbiome researchers, Astana, Kazakhstan

## Abstract

**Background:**

Alzheimer's disease (AD) is the leading cause of dementia and a critical social issue. Its multifactorial nature necessitates evaluating risk factors in diverse populations.

**Methods:**

This study analyzed 181 AD patients and 244 controls in Kazakhstan, comparing clinical, genetic, and microbial traits.

**Results:**

In our cohort, significant dementia‐associated variables included smoking, depression, dyslipidemia, insulin resistance, and liver dysfunction. AD patients had higher HDL, bilirubin, AST/ALT ratios, and lower ALT. Genetic analysis identified 13 SNPs linked to AD, notably in APOE, TOMM40, and MED12L genes involved in lipid metabolism, mitochondrial function, and gene transcription. APOE4 increased AD risk 1.9x, with higher prevalence in northern Kazakhstan (Astana). We also found specific alterations in the gut microbiome, specifically, a decreased Firmicutes/Bacteroidetes ratio, a reduced Bifidobacterium, and increased proteobacteria and inflammatory bacteria. The investigation of cytokine profiles demonstrated that pro‐inflammatory cytokines such as IFN‐γ, IL‐6, TNF‐β, MCP‐1, and IL‐17A were significantly elevated in AD patients, along with anti‐inflammatory cytokines IL‐4 and IL‐1RA, suggesting a dysregulated inflammatory response in AD. Additionally, elevated serum adiponectin levels, observed at three times higher than in controls, were strongly correlated with multiple cytokines and specific microbial taxa, such as Actinobacteria and Acidomicrobiia, indicating a potential interplay between gut microbiota, adipose tissue, and neuroinflammation in AD.

**Conclusion:**

These findings underscore the importance of considering bio‐geographic and environmental factors in AD research. The study's outcomes may aid in further research and the development of personalized approaches for managing and treating AD in distinct geographical regions.

Research support: Nazarbayev University Collaborative Research Program Grant [Funder Project Reference: 20122022CRP1602] and the Ministry of Higher Education and Science of the Republic of Kazakhstan Grant [Funder Project Reference: AP14871338].

